# Conducting school-based health surveys with secondary schools in England: advice and recommendations from school staff, local authority professionals, and wider key stakeholders, a qualitative study

**DOI:** 10.1186/s12874-023-01957-x

**Published:** 2023-06-15

**Authors:** Lorna M. Hatch, Emily C. Widnall, Patricia N. Albers, Georgina L. Hopkins, Judi Kidger, Frank de Vocht, Eileen Kaner, Esther M. F. van Sluijs, Hannah Fairbrother, Russell Jago, Rona M. Campbell

**Affiliations:** 1 Population Health Sciences, Bristol Medical School, University of Bristol, Canynge Hall, Bristol, BS8 2PL UK; 2 Faculty of Medical Sciences, Newcastle University, Newcastle Upon Tyne, UK; 3 MRC Epidemiology Unit, School of Clinical Medicine, University of Cambridge, Cambridge, UK; 4 Health Sciences School, University of Sheffield, Sheffield, UK; 5 Centre for Exercise Nutrition & Health Sciences, School for Policy Studies, University of Bristol, Bristol, UK

**Keywords:** Health, Well-being, Young People, Students, Schools, Research, Recruitment, Retention

## Abstract

**Background:**

Improving the health and well-being of young people is a public health priority. Schools present an ideal setting to implement strategies to improve young people’s health and well-being. A key strategy involves conducting surveys to assess student health needs, inform interventions, and monitor health over time. Conducting research in schools is, however, challenging. Schools can find it difficult to participate and adhere to research processes, even when they are keen to be involved in research, because of competing priorities (e.g., attendance and educational achievement), as well as time and resource constraints. There is a lack of literature on the perspectives of school staff and other key stakeholders working in young people’s health on how best to work with schools to conduct health research, and in particular, health surveys.

**Methods:**

Participants (*n* = 26) included members of staff from 11 secondary schools (covering students aged 11–16 years), 5 local authority professionals, and 10 wider key stakeholders in young people's health and well-being (e.g., a school governor, a national government member), based in South West England. Participants took part in semi-structured interviews that were conducted either over the phone or via an online platform. Data were analysed using the Framework Method.

**Results:**

Three main themes were identified: Recruitment and Retention, Practicalities of Data Collection in Schools, and Collaboration from Design to Dissemination. It is important to acknowledge the role of local authorities and academy trusts in the English education system, and work closely with these when conducting school-based health surveys. School staff prefer to be contacted about research via email and in the summer term, following exams. Researchers should contact a member of staff involved in student health/well-being, as well as senior leadership, during recruitment. Data collection during the start and end of the school year is undesirable. Research should be collaborative with school staff and young people, consistent with school priorities and values, and flexible and tailored to school timetables and resources.

**Conclusions:**

Overall the findings demonstrate that survey-based research methods should be school-led and tailored to each school.

**Supplementary Information:**

The online version contains supplementary material available at 10.1186/s12874-023-01957-x.

## Background

Improving young people’s physical and mental health has been identified as a key public health priority [1, 2]. Currently, 1 in 6 young people aged 5 to 17 years have a mental health disorder, a rise from 1 in 9 in 2017 [3]. Children’s emotional health impacts their cognitive development and capacity to learn [4, 5], with a bidirectional relationship reported between educational attainment and mental health [6]. Young people’s risk-taking behaviour (e.g., alcohol and substance misuse, sexual health, peer and intimate relationships) has also been highlighted as a public health concern [7]. Furthermore, recent data show that only 36% of 10–11 year olds meet physical activity guidelines [8, 9], while 50% are classified as overweight or obese [10]. These statistics are concerning as many health behaviours, diseases and disorders track into adulthood [11–14] and are major risk factors for several chronic conditions including Type 2 diabetes mellitus and cancer [15–18].

Although all young people may be at risk of poor health outcomes, significant health inequalities exist, with increased deprivation associated with a range of poorer health outcomes [19, 20]. These disparities have been exacerbated by the coronavirus pandemic, which has led to fundamental changes in the lives of young people [8, 21]. For example, while some research suggests that young people have coped generally well during the pandemic [22], other evidence emphasises that certain young people, including those who are disadvantaged economically (experiencing poor quality, overcrowded housing and food poverty) [23], have experienced a greater negative impact on their health and well-being [24]. The importance of early identification and intervention to improve young people’s health and well-being is well documented [25, 26]. Schools are a key public health setting as most young people attend school and spend a significant amount of their time there. Schools offer a valuable opportunity for engaging with a large and diverse sample of young people from different socioeconomic backgrounds. Schools have thus been encouraged to form partnerships with public health teams and researchers to improve the health and well-being of their pupils [24, 25].

Moreover, in 2021, Public Health England (now the Office for Health Improvement and Disparities) and the Department for Education identified eight principles for a whole-school approach to promoting young people’s mental health and well-being; a key principle was to assess needs and monitor impact of interventions [27]. This can be supported by school-based health research, which can promote health and well-being through implementing, monitoring, and evaluating interventions, and providing evidence-based recommendations for change. School-based health surveys, in particular, can enable rigorous and timely examination of need and monitoring of change on a local, regional, and national level; this data can be used to inform interventions aimed to improve health and well-being. However, barriers to improving health and well-being through school-based research remain.

Recruiting schools for research is challenging [28, 29]. Recruitment efforts do not always reach the staff who might be interested and/or qualified to engage [28]. Moreover, the education system in England has changed in the last few decades, with a substantial decrease in the number of local authority-maintained schools and rise in independent academy trust schools [30]. Now, only 20% of secondary schools in England are directly controlled by their local government with the remainder reporting to central government via semi-autonomous academy trusts [31]. This shift in landscape is likely to have weakened the recruitment pathways between researchers, local authorities, and schools. Academy schools have no obligation to follow the national curriculum (inclusive of health-related provision) and therefore the scope for influencing practices in relation to health has been diminished [32]. Evidence shows that there are differing practices between and within academy trusts towards student health promotion and protection [33]. As a standardised strategy is rare, approaches to student health are often delegated to individual academy schools and staff, with some staff not considering health promotion as a key function of academy schools/multi academy trusts [33]. Furthermore, while some schools (irrespective of status) are keen to participate in research to support pupil health, they perceive challenges due to a primary focus on education and academic attainment, and a lack of school time [34].

Given the individual approach to health promotion within schools, a better understanding of how to effectively conduct health research across varying school settings is needed. While a great deal of school-based research has been conducted, there is a lack of information on the processes of working with schools and the most effective means of collaborating with schools to improve the health and well-being of young people. Most of the literature that does exist, has focused on highlighting the basic principles of school-based research for novices in the area, and solely reports researcher perspectives on this through descriptive/commentary papers [35–37]. Moreover, there is a paucity of literature on the perspectives of school staff and other key stakeholders on how best to work in partnership with schools to conduct school-based health surveys. For example, there is a lack of evidence on school staff and key stakeholders’ opinions and preferences regarding recruitment and data collection procedures, and the factors which influence participation in survey-based research and adherence to research processes. This gap is important because research practice and impact could be improved through the insight of key stakeholders who understand the complexities of conducting school-based research from experience within the school and the wider system that schools operate in. Moreover, while a few papers have discussed the practicalities and challenges of conducting research with schools, these considered research more generally and were published in the US and are therefore based on the American education system [29, 38]. Although charter schools in the US operate in a similar way to academies in England, in that they act independently of the state schools system [39], US studies are not likely to represent the intricacies of conducting research in English schools, particularly with regard to the involvement of local authorities and multi-academy trusts.

The aim of this paper is to explore the perspectives of UK secondary school staff, local authority professionals, and other key stakeholders on how best to work with secondary schools to conduct school-based health surveys relating to young people; this includes factors which are considered to impact participation in research, and preferences regarding recruitment and data collection processes which can facilitate adherence. A further aim is to provide key practical and logistical guidance for collaborating with secondary schools and stakeholders for school-based health surveys with young people in schools. The specific research questions are:

1. What are the key factors perceived by UK staff and key stakeholders to influence a secondary school’s participation in health surveys for young people and adherence to the research processes?
2. What are UK secondary school staff’s and key stakeholders’ preferences and advice regarding recruitment and data collection processes?
3. How does academy status/affiliation and relationship with local authorities impact on participation in health surveys in English schools?

## Methods

### Participants and study design

The data reported in this paper were from a pilot study designed to establish a South West School Health Research Network (SW-SHRN) [40]. The aim of the SW-SHRN is to collect high quality data to inform school health policy, practice, and implementation, to improve the health, well-being, and educational attainment of school-aged children in the South West of England. The SW-SHRN pilot study commenced during the pandemic (April 2020) with recruitment ending in July 2022. The SW-SHRN pilot study involved cross-sectional survey data collection on the health, well-being, social connectedness, and risk-taking behaviours of secondary school children (in years 8 and 10, 12–15 years of age) across 7 local authorities in the South West of England. Each participating school received an individualised school report which summarised their students’ data benchmarked against average data for all schools in the network. School environment questionnaires were also completed with a member of staff from each school to develop a regional picture of existing school health policies and programmes. Semi-structured interviews were completed with key school contacts in participating schools, local authority professionals, and wider key stakeholders working in young people’s health and well-being to investigate their experience of participating in this School Health Research Network pilot study, and to gather advice on how to improve school-based health research of this nature, with a particular focus on school-based health surveys. The findings from these qualitative interviews are the focus of this paper.

A member of staff from each school participating in the SW-SHRN pilot study (*n* = 18) was invited to participate in an interview; a staff member with a health/well-being role was requested and an appropriate individual was identified by the school. A member of staff involved in young people’s health from each local authority in South West England (*n* = 7) was invited to participate. Wider stakeholders which were invited to participate (*n* = 13) were pre-identified by the project team at the point of study design as individuals within England with a key role relating to child health and well-being; this included individuals working in national government, higher education, and charities. Nine individuals (one key stakeholder, two LA professionals, and six school staff members) did not respond to the invitation. Three individuals (two key stakeholders and one school staff member) declined the invitation; reasons for this included no longer working within young person health and lack of capacity*.* A total of 26 participants volunteered to participate in an interview; this included members of staff from 11 schools, 5 local authority professionals and 10 wider key stakeholders in young people’s health, based in England. School staff roles varied and included senior leadership, subject leads (e.g., Personal, Social, Health and Economic education [PSHE] leads) and more specific health and well-being roles. Wider key stakeholder organisations/individuals consisted of a charity (*n* = 1), university researcher/clinician (*n* = 1), academy trust governor (*n* = 1), the NHS (*n* = 1) and various government departments (*n* = 6).

Interviews took place remotely either over the phone or via an online platform (e.g., Microsoft Teams). For the purposes of this paper, the questions utilised from the overarching interview topic guides (see supplementary files 1 and 2) were centred around how best to work with schools for school-based health research, with a particular focus on health surveys.

The University of Bristol Faculty of Health Sciences Research Ethics Committee granted ethical approval for this study (Ref. 110922). School staff, local authority professionals and wider key stakeholders were approached by a member of the research team via email and invited to participate in a semi-structured interview. Participants received an information sheet and were given an opportunity to ask questions. All participants provided written informed consent in advance of the interview.

### Data analysis

NVivo version 12 (QSR International) software was used for data management and to assist data analysis. The Framework Method was utilised to analyse the data [41]. The Framework Method involves a systematic approach which enables the identification of similarities and differences within qualitative data; it involves the examination of relationships between different parts of the data, thereby supporting descriptive and/or explanatory conclusions, which are clustered around themes. Data were compared across cases (participants), as well as within cases. The Framework Method was considered the most appropriate approach to data analysis for several reasons. Firstly, the structured step-by-step analysis process is useful when multiple researchers are working on a project, and for managing large data sets where deriving a holistic overview of the entire data set is necessary, both of which were the case in this project. Additionally, the Framework Method is flexible and not aligned with a particular philosophical, epistemological, or theoretical approach [41]. The Framework Method was therefore appropriate as the area of investigation is not underpinned by theory. Moreover, the charting process within the Framework Method enabled the data to be explored and described at an organisation level (e.g., school, local authority, government department). Furthermore, the Framework Method is characterised by reflexivity, rigour, and quality and is now used widely within health research [42–50]. The Framework Method involves seven distinct stages: transcription, familiarisation with the interviews, coding, developing a working analytical framework, applying the analytical framework, charting data into the framework matrix, and interpreting the data [41]. For a full description of the Framework Method, including how each of the seven stages were applied to this project, please refer to supplementary file 3.

## Results

Participants were key stakeholders working in young people’s health. Participant characteristics, including job role and employer organisation type, are presented in Table 1. School locations included urban (*n* = 4), rural (*n* = 4) and coastal (*n* = 3). Free school meal eligibility status (used as a proxy for socioeconomic status) at participating schools ranged from 1.3% to 50.1%. The 2022 English average free school meal eligibility is 22.5% [51]. The Ofsted rating of participating schools varied and included outstanding (*n* = 2), good (*n* = 5), and requires improvement (*n* = 2); two were new schools and had not yet received an Ofsted rating.

**Table 1:** Summary of school staff and key stakeholder interviews by organisation and role type

Interview	Organisation Type	Role Type/Department
KS1	Charity	Mental health lead
KS2	Government department	Mental health, national
KS3	Government department	Public health, national
KS4	Government department	Public health, regional
KS5	Government department	Research lead, national
KS6	Government department	Public health, national
KS7	University	Clinical Psychologist/Academic
KS8	Academy Trust	Governor
KS9	NHS	Mental Health Support Team
KS10	Government department	Mental Health, regional
LA1	Local authority	Health & Wellbeing
LA2	Local authority	Children & Young People
LA3	Local authority	Children & Families Commissioning
LA4	Local authority	Children & Young People
LA5	Local authority	Children & Families
SC1	Academy school	Deputy Head Teacher
SC2	Academy school	Pastoral Support Worker
SC3	Local authority-maintained school	Deputy Head Teacher
SC4	Local authority-maintained school	Head of Personal Development Curriculum
SC5	Academy school	Deputy Head Teacher, Student Welfare & Behaviour
SC6	Local authority-maintained school	Music Teacher, Lead for Looked After Children
SC7	Academy school	Mental Health & Wellbeing Coordinator
SC8	Local authority-maintained school	PSHE Lead
SC9	Academy school	Assistant Headteacher
SC10	Local authority-maintained school	Deputy of PE and Health, PSHE Lead
SC11	Academy School	Deputy Head Teacher

‘*KS*’ key stakeholder, ‘*LA*’ Local Authority ‘*SC*’ School Contact

This section provides summaries for each theme and sub-theme produced, with exemplar quotes. Table 2 presents an overview of the three main themes, and corresponding sub-themes, developed. The data presented below was collected as part of a wider study which involved a school-based health survey, however in some instances participants discussed opinions and preferences regarding school-based health research more broadly.

**Table 2:** Themes and sub-themes related to working effectively with secondary schools for health surveys

Theme	Sub-themes		
Recruitment & Retention	Priorities & Values	Contextual Challenges: Local Authorities vs. Academy Trusts	Recruitment Recommendations
Practicalities of Data Collection in Schools	Complexities of Consent	Working Flexibly with Schools	Researcher-in-the-Room
Collaboration from Design to Dissemination	Schools Shaping Research	Student Voice	

### Recruitment & Retention

This theme captured participants experiences and recommendations for how to maximise schools’ engagement with health research more broadly, and centres on understanding schools’ drivers and individual contexts. Three sub-themes were apparent in the data: Priorities & Values; Contextual Challenges: Local Authorities vs. Academy Trusts; and Recruitment Recommendations.

#### Priorities & Values

This sub-theme reflects the tension that exists between academic criteria and health and well-being. Stakeholders noted that schools are often focused on academic achievement and attendance, despite many stating that their school culture centres around health and well-being. Schools have limited resources (e.g., time and money) which are typically allocated towards curriculum learning.

“Obviously their priority is attainment and attendance because that is their bread and butter and that’s what schools do. But we know that schools want to support mental health and well-being” (KS 1).

School staff highlighted that when a university is reputable it acts as a motive for engagement in health research. Moreover, school staff and local authority professionals explained that schools may want to be recognised for investing time in student health and well-being.

“I think also…being able to showcase them as a school. Because I think some schools really want to be celebrated for the work they do. And I think now, especially maybe the academies as well, they're always looking at ways that they can show why parents should send their kids to that school, why kids should want to come.” (LA1).

#### Contextual Challenges: Local Authorities vs. Academy Trusts

A challenge in England is the separation of the school system into local authority-maintained schools and academies, who act independently. Stakeholders, local authority professionals, and school staff discussed the relationships between local authorities, schools, and academies and whether they thought this would impact a school signing up to school-based health research.

“I think, getting into schools has become a lot more challenging just because of how they’re set up and the academy structure. It used to be that you could go through the local authority and have a quite straightforward way of getting into schools because they were quite linked up with what they were doing. I think now that…has broken down and… it’s very much up to the individual schools, or certainly the academies, whether they want to engage or not.” (KS 6).

An overall message was that gaining approval from the entity providing support to a school, whether that be an academy trust or local authority, may be important when it comes to a school’s decision to participate in research. However, while approval from an academy trust was considered particularly important for recruiting an academy, local authority-maintained schools differed in their relationships with their local authority and opinions regarding how this influenced recruitment. Some schools work closely with their local authority and look to them for recommendations regarding health programmes, practices and research to get involved in. Other schools are more disconnected from their local authority and suggested that approaching the school directly would be most effective for recruitment to research.

“I think going through the local authority can be the slower process just because they’re so busy. Especially our one, it seems to be very understaffed at the moment so it can be difficult to receive communications such as this from them, so…going straight to the senior leaders is a better way.” (SC 6).

#### Recruitment Recommendations

Participants offered advice on effective recruitment of schools, including which member/s of staff to contact, the preferred methods of contact, and when during the academic year schools are most likely to engage in research. School staff and key stakeholders recognised the difficulty in recruiting schools due to staff turnover/changing roles, the differing levels of investment in health and well-being from senior leadership, and the lack of time and resource for schools taking on additional projects. Schools discussed the importance of senior leadership support as they make the final decision on participation, but detailed that making initial contact with a member of staff invested in health/well-being (e.g., mental health and well-being coordinator, PSHE lead) may be beneficial, as they can encourage senior leadership to participate.

“Whereas you never know do you. If you’re sending it to the head, the head might be, ‘Oh, I'm not getting involved in this’, but actually the person who is the expert, shall we say, might think, ‘Well actually, that is a really good thing. That would really help me with what I'm doing.’ So, they might just think that the obvious way in is through the head, because otherwise it is a bit of a faff for yourselves, getting hold of the right person.” (SC 5).

There was consensus that the preferred method of contact involves receiving an initial email with details of the study and then a follow up email and/or call over the following few weeks. Additionally, one member of school staff highlighted that recruitment efforts were most likely to be successful if initial contact with the school is made in the period following summer exams, as staff are planning the curriculum for the following year and have more flexibility to incorporate a research study into the school timetable. Although school staff differed in opinion regarding the time of year that schools are most likely to accommodate research, there was a consensus that the start of the school year (September) and the summer term (June-July) was least likely due to a focus on integrating new students and exams, respectively.

“So from…about April time, it’s Year 11 exams. I think probably September time it's integrating the new Year 7 s, so probably, I think, January would be a good time [for participation in data collection], probably avoiding more towards the end of the year.” (SC 9).

### Practicalities of Data Collection in Schools

This theme centers around practical advice from school staff, local authority professionals, and wider key stakeholders on collecting survey-based health data in school and was split into three subthemes: 1) Complexities of Consent; 2) Working Flexibly with Schools and 3) Researcher-in-the-Room.

#### Complexities of Consent

School staff provided varied views on the consent process, particularly regarding whether parents/carers needed to provide consent for their child to participate as well as the child providing individual consent, and whether this differed among age groups. There was a concern that some students may not fully understand the research project and/or what would be required of them, and therefore would not be able to make an informed decision. As such, some school contacts felt that parental/carer consent (as well as child consent) should be obtained, particularly as they are the legal guardian/s while the students are under 16 years of age. However, other school contacts felt that it should be exclusively the student’s choice whether they participate in research if they are secondary-school age.

“The parent might say no and then the student might say yes. I think for under 16 s particularly, I think the parent one trumps the student one. Only because they’re legal guardian. If they’ve read through the documentation, they might not be happy, whereas a student who might not understand fully what it is and they want to do it, I guess you’d have to go with the parental decision.” (SC 6).

“I would say that your average secondary school age pupil should be able to give informed consent… Ethically I don’t see a problem with that.” (SC 13).

One member of school staff expressed different views on consent, depending on whether they were speaking as a staff member or parent.

“As a parent, and I’ve got a Year 9 daughter, I think she’d be more than capable of making a decision about what to ask her questions on. It is slightly different with Year 8. I think it would be important to let parents know that that’s what you’re doing.” (SC 13).

Although there were some differences in opinion regarding child versus parent/carer and child consent, there was consensus among school staff regarding parent/carer opt-out versus opt-in methods for school-based health surveys. An opt-out method in this instance would involve all children being automatically enrolled to a study and a parent/carer having to contact the school/researchers to opt their child out of the study if they do not want to participate. In contrast, an opt-in method would involve parents/carers having to contact the school/researchers to enroll their child in a study. School staff agreed that the opt-out method is preferable to the opt-in method, due to efficiency. Moreover, they expressed a preference for researchers to manage this process in order to reduce burden on schools.

#### Working Flexibly with Schools

School staff had different preferences around the logistics of conducting surveys in schools. This included which lesson/s to use for data collection, as well as the amount of curriculum time data collection requires. Several agreed, however, that if the research project is health-based then fitting data collection into personal, social, health, and economic (PSHE) lesson time felt the most appropriate and least disruptive. The overarching message from schools was that the researchers must be flexible in their approach to schools based on their individual preferences and must not expect schools to adapt to meet research demands. While reflecting on data collection for survey-based research, one participant expressed a preference for being able to book data collection sessions flexibly and sporadically over a number of weeks, at times which would best suit the school, their students and the timetable.

“If you were to say to me, ‘Here's my team of three people. You've got them for two weeks,’ and I'd be like, ‘I want them here, here, here, here, here,’ which is annoying because it's really sporadic. It’s like three hours on a Thursday afternoon, but in terms of if you're asking me what's best for my students…that’s what’s best for them.” (SC 1).

#### Researcher-in-the-Room

The majority of schools had a preference for in-person, researcher-led survey data collection. They felt that having a researcher present encourages interest and engagement in the research and ensures consistency in data collection procedures. However, one school contact said that they did not see the benefits of a researcher leading data collection if it involved a protocol they were familiar with (i.e., administering a survey), particularly if a member of school staff still needed to be present in the lesson to manage registering students and behaviour. School staff felt that response rates would be much lower if data collection was completed online at home (as opposed to in person) and may exclude certain students (e.g., disadvantaged students, those with special education needs [SEN]), and lead to response bias.

“I think having researchers there is massively beneficial, because I think it gets the students excited, because it’s like, “Oh, this is something different, I have not seen yourself before”. They're almost a little bit more invested, paying a little bit more attention. And I think obviously going into a bit more detail, maybe explaining- because obviously you're running it and it’s your project, you’ve got that bit more understanding as to why we’re doing what we’re doing” (SC 7).

“We get a much, much lower uptake when we’re accepting things remotely. I know that you probably would lose some groups. So, a lot of SEN students might not feel confident doing it without a bit of support. Maybe some of our disadvantaged students wouldn’t do it. I know that girls tend to complete homework more than boys. I think you would lose…your data would become quite skewed.” (SC 8).

### Collaboration from Design to Dissemination

This theme refers to the importance of working closely with schools and young people throughout all stages of the research process, there were two subthemes: 1) Schools Shaping Research and 2) Student Voice.

#### Schools Shaping Research

Stakeholders emphasised the importance of listening to school needs and seeking to understand schools’ experiences of participating in research. One school contact felt that for research to be effective researchers needed to have a comprehensive understanding of the education sector. Moreover, participants felt that researchers should seek guidance from school staff when shaping research study materials and during dissemination.

“The one thing that I would say is it needs to come from a standpoint of understanding the educational sector. Oh, I'm not trying to be patronising, but sometimes we've had information given to us by organisations which is all very well intentioned, but they're not teachers, and actually understanding how the information can be presented to students or what is appropriate is very different.” (SC 4).

#### Student Voice

School staff, local authority professionals, and wider stakeholders advised involving young people in the research project, from the set-up of the study through to dissemination of findings. Participants felt that young people should be involved in decision making processes and the design of research materials, so that they are able to express their needs, priorities, and preferences. Additionally, one stakeholder suggested training young people to support researchers with research data collection to empower students in the research process. This was thought to be mutually beneficial, as students would develop research skills and researchers would have support with data collection, as well as greater buy-in from students.

“Let us co-own [research] with young people in schools and let us really make these decisions together about what we collect and how…give them chance to vote on priorities…then I think you have got the buy in from them.” (KS 7).

**Fig. 1:**
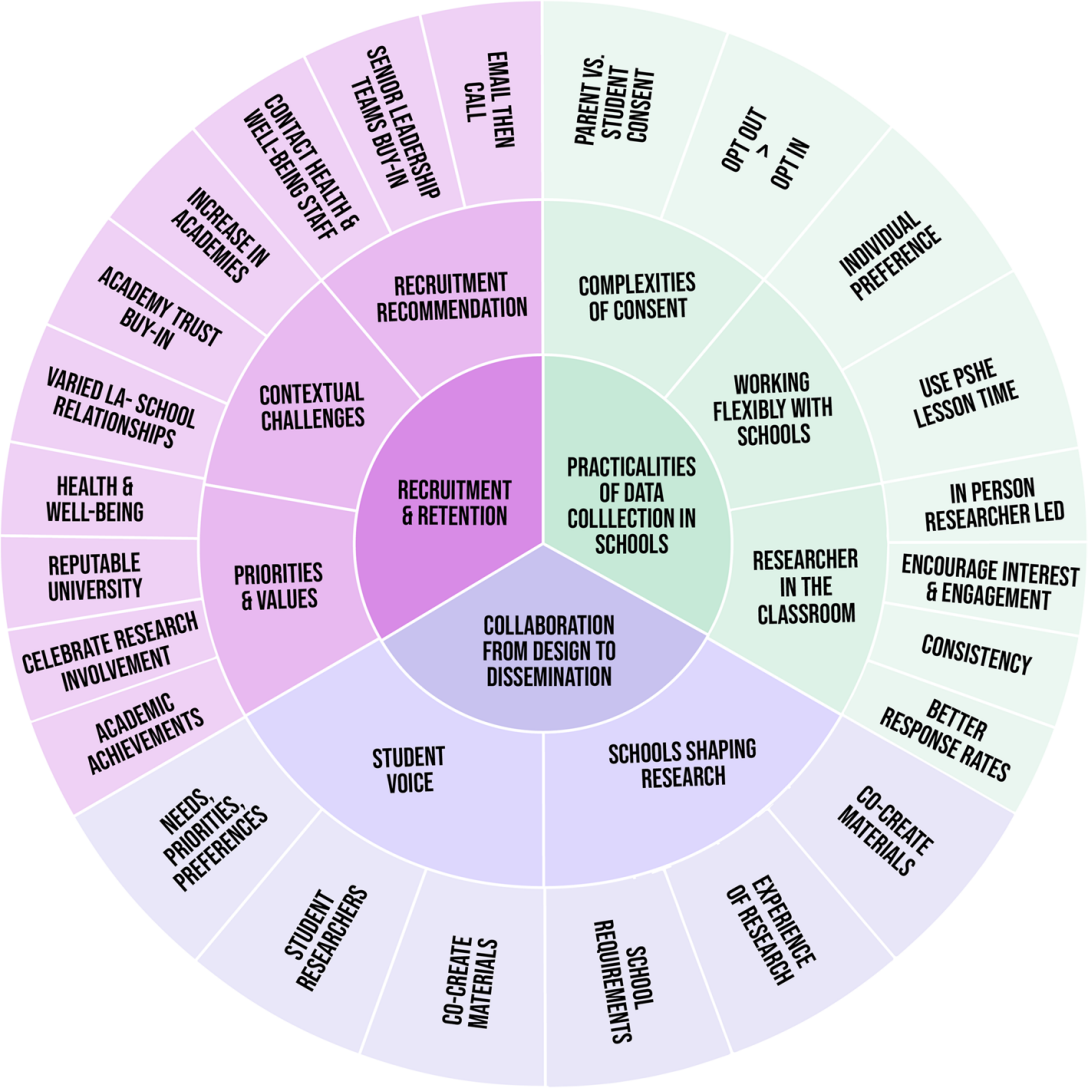
A visual diagram of effectively conducting health research in schools based on themes and subthemes. NB: ‘LA’ = Local Authority; PSHE = Personal, social, health and economic

By combining the themes, subthemes and context from participant quotes, Fig. 1 provides an overall visual representation of conducting school-based health surveys.

## Discussion

This study presents primary qualitative data on how to work effectively with schools on survey-based research to improve health outcomes in young people. As well as providing much needed insight into the complexities of conducting health surveys in schools, the findings demonstrate core opinions and preferences regarding school-based health research more broadly. The findings combine the views of a range of key stakeholders including school staff, local authority professionals, and wider stakeholders (e.g., national government members) and capture novel information on working with schools within the English education system.

Overall, the findings of this study indicate that to be successful, school-based health surveys must be school-led. Researchers must be flexible to meet school needs and priorities, as well as preferences regarding recruitment and data collection. Researchers must provide each school with an offer that works for them, not a rigid system that may be inaccessible to many schools. Moreover, researchers should seek to consult with schools and young people throughout the research process; this could be achieved through working with school councils and/or having a member of school staff and young person on a project advisory board to support bid development, as well as project implementation and dissemination.

In the present study, the majority of school staff expressed a preference for researcher-led data collection. Some schools may be willing to adopt teacher-led survey administration using standardised written instructions. This approach is used in the Health Behaviour in School-aged Children (HBSC) study, a cross-national survey on adolescent health and well-being undertaken every four years in over 41 countries [52]. A teacher-led approach may be more feasible and time- and cost-efficient for the research team, particularly for large-scale projects. However, it is important to recognise the additional burden this may place on teachers and that the protocol involved in a study will influence whether a school decides to participate. Researchers involved in the HBSC study note that when financial resources are available, researchers will administer the survey to minimise teacher burden [52]. Therefore, as aforementioned, research should be collaborative and school-led, and researchers should work with schools on an individual basis to facilitate their preferences (when feasible), as this will support recruitment and retention to the study.

The diverging opinions of stakeholders regarding the research consent process for school-based health surveys was an interesting discovery. While some felt that child only consent would be sufficient for children of secondary school age, others felt that consent from parents/carers (as legal guardians to those under 16 years old) was also necessary. There was a concern among some participants that young people may not fully understand the research project and what participation would involve. It’s important to note, however, that these findings reflect adult perspectives on adolescent understanding. In a study which explored adolescent (14–17 years) perspectives on participation in an alcohol intervention, many of the adolescents were reported to understand their rights as participants, assess possible implications of research participation and weigh up a decision about participating [53]. Moreover, there was no evidence that they felt they should seek approval or guidance from parents/carers when deciding whether to participate and no indication that they felt ill-equipped to make the decision alone [53]. In contrast, there was evidence that some adolescents did not have a full understanding of the study design, but shortfalls in understanding are not uncommon in research with adult participants [54]. The authors noted that much of the adolescents’ understanding of the research and research involvement was gained from verbal explanations provided by research staff, rather than the written information sheets. These findings demonstrate the importance of providing information in a clear succinct manner and offering opportunities for participants to seek clarification to inform their decision making through discussions with researchers; this applies to both parental/carer and child, and child-only consent process. There was consensus among participants in the present study that if parental/carer consent is sought, an opt-out consent process, that is managed by the research team, is most desirable.

Table 3 presents 10 key recommendations for working with schools to support recruitment and retention, and promote efficient and effective health research.

**Table 3:** Key recommendations related to themes for working effectively with schools to conduct health surveys

Theme & sub-theme	Research recommendations
**Recruitment & Retention**	
Priorities & Values	Ensure research project aims reflect school priorities and values. Highlight that this is the case upon recruitment. For example, explain how the project may encourage much needed improvements in student health and well-being, and emphasise how this can have a positive impact on attendance and academic achievement.
	Provide eligible schools, local authorities and academy trusts with evidence of the University/researcher’s reputation and expertise in the project area e.g., university league tables, impact factors, published work.
	Enable schools to celebrate and publicise their involvement in health research e.g., through an accreditation or logo. This offering, if available, should be communicated during the initial stages of recruitment.
Contextual Challenges: Local Authorities vs. Academy Trusts	Recognise the role that both local authorities and academy trusts play in the current English education context. Understand the need to involve and engage both parties for school-based health research. Acknowledge that the relationship between a school and local authority may differ between schools, and that this can determine whether a school will accept recommendations from their local authority with regards to participation in research.
Recruitment Recommendations	As well as the senior leadership team, seek out the contact details for a member of school staff directly involved in health and well-being (e.g., mental health, PSHE or safeguarding lead), as they can endorse the project to senior leadership internally.
	Aim to commence recruitment of schools following the summer exam period (May–July), make initial contact via email and follow up with a phone call to maximise recruitment success.
**Practicalities of Data Collection in Schools**	
Complexities of Consent	If possible, gain ethical approval for an opt-out consent method. Discuss with schools how to manage this process so it is least burdensome for them.
Working Flexibly with Schools	Work flexibly with schools, adapt to their individual needs and preferences. For example, the timing and specific location of data collection.
Researcher-in-the-Room	Choose in-school in preference to online/at home data collection methods and consider having researchers present in school to lead data collection, when feasible.
**Collaboration from Design to Dissemination**	
Schools Shaping Research & Student Voice	Consult with school staff and young people in the designing and shaping of research.

Actioning these recommendations will promote the development and maintenance of partnerships between schools and researchers. It will lead to greater success in recruitment efforts, and streamline data collection processes. Overall, this will help to support much needed improvements in young people’s health and well-being through school-based health research. The findings of this study support previous literature which has highlighted the importance of reciprocal learning [55] and a flexible approach when working with schools [35]. Some of the findings of this study mirror those of process evaluations highlighting barriers and facilitators for adoption and implementation of school-based interventions; specifically, research discusses the negative impact of time constraints [56], and highlights the importance of aligning an intervention with school philosophy and goals [57] and co-creating research activities with school staff [58]. Importantly, this study builds on previous work by elucidating how researchers can co-learn and work flexibly with schools. The study also provides novel practical advice for recruiting and retaining schools, and first-hand knowledge on the intricacies of working with schools within the complex English education system.

It is important to note that there may be some instances where researchers experience challenges to implementing these recommendations. For example, it may not always be possible to reflect each school’s priorities and values, or contact a member of staff related to health directly due to a lack of access to contact details. Given the parameters and resource constraints that schools (e.g., funding, staffing, time) and researchers (e.g., funding timescales, ethical review) experience, collaborating with schools and young people throughout the research process may not always be possible; researchers should thus consult with schools and young people where feasible and take creative approaches to reduce the burden on all parties. However, in light of the release of the 2019 Department for Education statutory guidance on Relationships, Sex, and Health Education for all schools in England [59], schools may be more willing to engage in, and dedicate resources towards, school-based health research. For example, schools may participate in health surveys to identify need and inform teaching in this area, as well as monitor meaningful change. Nevertheless, researchers will need to balance the preferences of schools and stakeholders with the requirements associated with conducting research; this includes gaining approval from a research ethics committee (e.g., regarding consent processes) and balancing flexibility with consistency in data collection methods. Therefore, the recommendations provided within this paper should be used as a guide for researchers, but may need to be adapted based on the specific aims, design and/or resources of a project.

While there are many strengths of this study, such as including the views of a wide range of stakeholders and the diversity of participating schools, the study is not without limitations. The school staff who participated in the study were situated in state schools and academies; the perspectives of staff from independent, free and faith schools may not be represented. Moreover, the data for the study were from a project on a School Health Research Network that collects survey data on the health and well-being of year 8 (12–13 years old) and 10 (14–15 years old) students. Therefore, while it is likely that the interviewee data are translatable to conducting school-based health surveys with young people of secondary school age (11–16 years old), recommendations and best practice for working with primary schools and/or colleges may differ. Additionally, as this was an opportunistic paper positioned within a broader process evaluation of a School Health Research Network using survey-based data collection, the data was confined to topic guides designed for that study. However, lots of practical advice came through from participants about working with schools to conduct health research more broadly. Nevertheless, additional recruitment and/or data collection complexities that are exclusive to school-based intervention or clinical studies, for example, may not be fully represented within this paper. Furthermore, as the school staff participants worked in schools that were already engaged in a health research project, their views may not be representative of staff from all schools, including those that do not engage with health research.

Future research should seek the perspectives of academy trust members and young people as they both play an important role in school-based health research. The role of parents/carers in school-based research was not a key focus of this paper (beyond their role in consent processes), however it would be valuable for future research to consider their involvement as key stakeholders and explore their views on school-based research processes; this includes their opinions on using curriculum time for health surveys and who should play a part in shaping the surveys. Furthermore, additional research which explores the transferability of these findings to other education systems (such as nursery and primary schools, private schools, and SEN schools) would be valuable.

## Conclusions

The health and well-being of young people is a public health concern, with rates of mental ill-health, obesity and physical inactivity at an all-time high. Schools are recognised as a key setting for improving health and well-being outcomes in young people. However, schools experience competing priorities as well as time and resource constraints. This makes recruitment of schools for health research challenging. This study reported the perspectives of school staff, local authority professionals, and several other key stakeholders in young person’s health on how best to conduct health research with schools in the UK. This paper provides a set of key practical recommendations for researchers conducting school-based health research which have the potential to improve future research practice within schools. These recommendations include that researchers must acknowledge the role of both local authorities and multi-academy trusts in the English education system, and work closely with both when conducting school-based health research. Research should be collaborative with school staff and young people, flexible and tailored to school timetables and resources, and aligned with school priorities.

## Supplementary Information

**Additional file 1:** **Supplementary file 1.** SW-SHRN School Contact Interview Topic Guide.

**Additional file 2:** **Supplementary file 2.** SW-SHRN Key Stakeholder Interview Topic Guide.

**Additional file 3:** **Supplementary file 3.** The Framework Method – additional information on the analysis approach utilised.

## Data Availability

The datasets used and analysed during the current study will be available from the University of Bristol data archive. To request the data from this study, contact the corresponding author Dr Lorna Hatch lorna.hatch@bristol.ac.uk.
