# Supplementary material for: Conducting school-based health surveys with secondary schools in England: advice and recommendations from school staff, local authority professionals, and wider key stakeholders, a qualitative study
Source: BMC Med Res Methodol. 2023 Jun 15;23:142. doi: 10.1186/s12874-023-01957-x (PMC10273669; doi:10.1186/s12874-023-01957-x)
Supplement: Supplementary file 1 — Additional file 1: Supplementary file 1. SW-SHRN School Contact Interview Topic Guide. [file 12874_2023_1957_MOESM1_ESM.docx]

**Supplementary File 1: SW-SHRN School Contact Interview Topic Guide**

**Background**

1. What is your role within the school?
   1. How did you (personally) come to be involved in the Network?
   2. Did you have authority over whether your school participated in the Network or did you have to consult with other staff/senior leadership team (SLT) members?
2. Could you briefly describe your understanding of the Network?
3. What was it that interested you and/or your school in participating in the Network?
   1. How did being part of the Network align with your school’s priorities and culture?
   2. Your school was offered a small gratitude payment of [£200 update] for taking part, how did you feel about this - was this an incentive to take part?

**Logistics**

1. What did you think about the way your school was recruited to the Network?

PROMPT: We contacted the school via email and sent an information brochure containing details of the study and Network.

- 1. How helpful was the information you received about the study?

PROMPT: Specifically the School Information Brochure.

- 1. How can we improve the recruitment process, what would be helpful for recruiting schools?
     1. Are there barriers to taking part that we should be considering?
     2. When is the best and worst time of year to be approaching schools about joining the Network?

1. What are your thoughts on the Network website?
   1. If they have not accessed the website: What information would you like there to be on the website?
2. A student can be opted-out of the study by themselves or by a parent/guardian. If the student is opted-out of the study by a parent, but the student wishes to take part, what do you think should be the course of action?
3. We collected student data in your school [delete options as appropriate]:

- In school/class with an external researcher present to lead data collection (using either school computers, researcher tablet devices or a combination of both)

OR

- In school/class with a teacher administering data collection

OR

- At home data collection during an online learning session and in class with vulnerable children

What were your experiences of data collection with the students?

1. Can you think of anything that may improve this process?
2. What would be your preferred method of student data collection post-pandemic?

*If interviewee completed the* *staff questionnaire (school environment survey) themselves…*

1. How did you find the questionnaire you completed for the study?
   1. What changes would you make to the questionnaire to improve it, if any?
      1. Did you feel any questions were missing that you would have liked to receive data on, if so, what were these?
   2. What were your thoughts on how you completed it – i.e., over the phone with a researcher?

**Being part of the NETWORK**

1. What benefits are you hoping to get out of being part of the Network?

PROMPT: Obtaining data about your students, having support to make changes to benefit your students’ health and wellbeing, newsletters, training courses or outreach days.

- 1. What would you like to see from the Network, aside from the feedback report and meeting to discuss the findings of the school surveys?

*If the interviewee has not yet received their student report…*

1. You will soon be receiving your student health and well-being report with the results from the student questionnaires that the Year 8s and 10s completed as part of our research. What kind of information would you like to see in the report?
   1. How would you like this presented?
2. The second report you receive will benchmark your schools’ data to average data from all participating schools, what are your thoughts on this?
   1. Is there any particular information or schools you wish to be benchmarked against?
3. What do you hope to gain from the 1:1 meeting that is planned with the senior researcher to discuss the findings?
4. What do you aspire to do with the feedback we provide you?

PROMPT: Think about potential changes you could make to school policies and interventions

- 1. Do you envisage needing any help or support to carry out any plans?

*If the interviewee has received their student report…*

1. You received your first student health and well-being report with the results of the student questionnaires that the Year 8s and 10s completed as part of our research. Can you tell me what you thought of the report?

PROMPTS: Think about what and how the information was presented and the length of the report.

1. Are there any discriminators (e.g. ethnicity or disability) that you would be interested in having shown in the report?
2. How would you improve the report to ensure schools fully benefited from it?
3. The second report you receive will benchmark your schools’ data to average data from all participating schools, what are your thoughts on this?
   1. Is there any particular information or schools you wish to be benchmarked against?

*If they have not had their 1:1 feedback meeting:*

1. What do you hope to gain from the 1:1 meeting that is planned with the senior researcher to discuss the findings?
2. Have you taken any action as a result of the feedback we have provided you?

IF YES: What action have you taken?

IF NO: What do you aspire to do with the feedback we provide you?

PROMPT: Think about potential changes you could make to school policies and interventions

- 1. Do you envisage needing any help or support to carry out any plans?

*If they have had their 1:1 feedback meeting:*

1. How did you find your 1:1 meeting with the senior researcher to discuss findings?
   1. What was beneficial about it?
   2. How would you improve the meeting?
   3. Was there anything that did not need to be covered?
2. Have you taken any action as a result of the feedback we have provided you?

IF YES: What action have you taken?

IF NO: What do you aspire to do with the feedback we provide you?

PROMPT: Think about potential changes you could make to school policies and interventions

- 1. Do you envisage needing any help or support to carry out any plans?

1. What plans, if any, do you have to circulate or discuss the findings with any of your internal stakeholders, e.g., parents, students or governors?
2. Is there anything we could do or any materials we could provide you to aid your ability to integrate the Network into your school?

**Sustainability**

1. Would you be happy to participate in the next round of surveys within the Network?
   1. If not, can you think of why?
   2. What could encourage your continued participation in the Network?
2. How, if at all, do you think that academy status/affiliation could impact on whether schools take part in the network?
3. How, if at all, do you think whether a school’s local authority supports and participates in the network could impact on whether schools take part in the network?

**CLOSING**

- Is there anything else that you think is important that we should know about regarding what we have discussed today?
- Do you have any questions for me?
