# Supplementary material for: Conducting school-based health surveys with secondary schools in England: advice and recommendations from school staff, local authority professionals, and wider key stakeholders, a qualitative study
Source: BMC Med Res Methodol. 2023 Jun 15;23:142. doi: 10.1186/s12874-023-01957-x (PMC10273669; doi:10.1186/s12874-023-01957-x)
Supplement: Supplementary file 2 — Additional file 2: Supplementary file 2. SW-SHRN Key Stakeholder Interview Topic Guide. [file 12874_2023_1957_MOESM2_ESM.docx]

**Supplementary File 2:** **South West - School Health Research Network (SW-SHRN) Key Stakeholder Interview Topic Guide**

**Introduction for** **Local Authority (LA) contacts already participating in the project**

*Thank you for taking part in the interview today. As you will know, your local authority is collaborating with University of Bristol to create a new School Health Research Network with secondary schools in the South West of England. Some secondary schools in your area have taken part already. We are interested in your opinions on the Network and would like to discuss these with you.*

*This interview will broadly consider the following themes:*

1. *Your thoughts and opinions on the Network*
2. *How you can benefit from the Network*
3. *How you could potentially support the Network long-term*

**Introduction for non-LA stakeholders with prior knowledge of the project**

*Thank you for taking part in the interview today. You have been involved in research being carried out by the University of Bristol to create a new School Health Research Network with secondary schools in the South West of England. We are interested in your opinions on the Network and would like to discuss these with you.*

*This interview will broadly consider the following themes:*

1. *Your thoughts and opinions on the Network*
2. *How you can benefit from the Network*
3. *How you could potentially support the Network long term*

**Introduction for non-LA stakeholders with no prior knowledge of the project**

*Thank you for taking part in the interview today. I would like to explain a little bit about the School Health Research Network before we begin and have created a couple of slides to help me do this.*

[Share slide set with participants]*:*

*We have recently undertaken a pilot study involving the creation of a new School Health Research Network with secondary schools in the South West of England. For this we asked secondary school children to complete a survey about their health and well-being, and a member of school staff to report on health promotion policies and interventions delivered in their school. The aim is to revisit schools every two years and create a longitudinal dataset on schools in the South West. Each school receives a tailored report on the health and well-being of their students, and a summary report of health policies and interventions being delivered across schools participating in the Network. Each participating local authority also receives a summary report on the data collected from schools participating in their area. The primary purpose of the Network is to help improve health and wellbeing and educational attainment in schools in the network by creating a collaborative framework to facilitate a long-term relationship between University research and key stakeholders in public health delivery for school-aged children. I hope this provides some context on the Network for you. Do you have any questions?*

*This interview will broadly consider the following themes:*

1. *Your thoughts and opinions on the Network*
2. *How you can benefit from the Network*
3. *How you could potentially support the Network*

**Background on SW-SHRN**

Explain the Network further if needed and answer any participant questions.

**Stakeholder context**

1. Could you briefly describe your role and how it connects with school health and wellbeing?
   1. Do you have any direct influence over school health policies, interventions or commissioning? Please expand.

**About the SHRN**

1. Can you tell me your thoughts on the Network as a framework for improving school children’s health?
   1. What potential do you think the Network has?
   2. Any limitations / challenges to consider?
2. How do you think you and your organisation could benefit from the Network?

PROMPT: That could be partnership working, use of the data, activities beyond the survey…

1. How do you think the Network could be utilised to its full potential for schools and those working in the context of school public health?
   1. What services could the Network provide?

**Supporting the Network**

1. What would encourage you to buy into and support the Network?
   1. How about supporting the Network on a long-term basis?
2. How do you think you could support the Network in terms of:
   1. Recruitment of and access to schools
      1. How do you currently influence schools?
3. How do you think you could support the Network in terms of:
   1. Funding the Network
      1. Would you have capacity to financially support the Network?
      2. *If YES* – Under what conditions could you provide financial support?

*Note to interviewer*:

If the participant talks about or mentions collecting data from primary schools or early years (in response to this or any other questions), ask what the reasons behind this are.

- - 1. Could this be long-term support or a one-off contribution?

1. How do you think you could support the Network in terms of:
   1. Dissemination of findings to schools and other stakeholders (i.e., how can you facilitate the sharing of findings to ensure the outcomes reach a wide range of audiences)

**Network output**

1. What type of outputs would you like to see from the Network?

PROMPT: Blogs, newspaper articles, policy briefings, evidence summaries

1. How do you think the Network could evolve to deliver other resources to support wider children and young people’s health?
   1. How do you think stakeholders could use the findings to develop and implement health policies in school?
2. Health has recently become a compulsory part of the school curriculum. Expectations on the delivery of this curriculum may vary depending on school circumstance but should have begun teaching by the summer term of 2021.
   1. What are your opinions on health forming part of the inspection framework in England?
   2. How do you think this could influence schools signing up to the Network?
      1. How do you think the Network could support schools in relation to the new curriculum?

*For Local Authority participants who have not received their report…*

1. You will receive a report on the data from the schools in your area, specifically what kind of information would you like to see in the report?
   - - - 1. How would you like this presented?
2. The report, where appropriate, will also benchmark to other local authority data, what are your thoughts on this?
3. How useful would this be to you?
4. Is there any particular information you would like to be benchmarked against?

*For Local Authority participants who have received their report…*

1. You received a report on the data from the schools in your area, can you tell me what you thought of the report?

PROMPTS: Think about -

What and how the information was presented

The length of the report

The benchmarking data

1. How would you improve the report to ensure that local authorities could fully benefit from it?

**COVID-19**

1. How do you think the Network may have helped support schools throughout the COVID-19 pandemic, including their COVID-19 recovery response?

**Sustainability and scalability**

1. How do you think we could encourage schools to sign-up to the Network?
   1. How could we ensure schools remain part of the Network long term?
2. Because of the COVID-19 pandemic, where possible we gave schools the option of having a researcher present in class to manage the data collection process. How valuable do you think it is to have researchers lead data collection within a school and why?

**Closing**

- Is there anything else that you think is important that we should know about regarding what we have discussed today?
- Do you have any questions for me?
