# Supplementary material for: Conducting school-based health surveys with secondary schools in England: advice and recommendations from school staff, local authority professionals, and wider key stakeholders, a qualitative study
Source: BMC Med Res Methodol. 2023 Jun 15;23:142. doi: 10.1186/s12874-023-01957-x (PMC10273669; doi:10.1186/s12874-023-01957-x)
Supplement: Supplementary file 3 — Additional file 3: Supplementary file 3. The Framework Method – additional information on the analysis approach utilised. [file 12874_2023_1957_MOESM3_ESM.docx]

**Supplementary File 3: The Framework Method – additional information on the analysis approach utilised**

The Framework Method was utilised to analyse the data. The Framework Method involves seven distinct stages. Each stage is discussed below with reference to how these steps were applied to this project.

- Stage 1: Transcription: Audio recordings of each interview were collected. These were transcribed verbatim. The transcripts were then reviewed and checked for accuracy by EW.
- Stage 2: Familiarisation with the interviews: All transcripts were initially read by EW to gain familiarity with the data. Any preliminary contextual, analytical or reflective notes, thoughts or impressions were recorded. Preliminary notes took the form of early annotation of the interview transcripts; this included notes on the context of the interview (e.g., time of year, political context) and organisation-specific references (e.g., size and location of school, organisational structure).
- Stage 3: Coding: Initially, EW and LH independently read and annotated six transcripts; two school contact interviews, two local authority interviews and two wider key stakeholder interviews. The transcripts were reviewed line by line, with a descriptive label/paraphrase (‘code’) applied when a passage within the transcript was interpreted as relevant and/or important in relation to one or more of the research questions; a preliminary list of codes and draft framework was produced. The preliminary list of codes was data-driven and based on both researchers’ contextual, analytical and reflective notes collected during Stage 2. Examples of codes within the preliminary list of codes include ‘making contact’; ‘consent’; and ‘perceived benefits’. The draft framework consisted of the first full set of codes, which were built on in Stage 4 to develop a working analytical framework. Additionally, EW and LH gathered a holistic impression of what was said and of different perspectives within and between participants. For example, a holistic impression that came through the transcripts was that schools experience time and resource constraints which impact their involvement in research.
- Stage 4: Developing a working analytical framework: After coding the first six transcripts, EW and LH met to compare and discuss the initial codes, draft framework and holistic impressions. Consequently, EW and LH agreed on a set of codes, which were grouped into categories and clearly defined, to apply to all subsequent transcripts. This formed a working analytical framework. This process was iterative, codes from both researchers were compared and refined, and codes changed and developed over time. Where there was agreement between researchers, codes and categories were kept. When there was heterogeneity between researchers, these codes/categories were discussed extensively and either kept or merged with an existing code or category. Moreover, codes were included to cover variation in the data. While there were some distinct differences between school contact interviews and wider stakeholder interviews, sufficient overlap existed to enable all transcripts to be coded within the same analytical framework.
- Stage 5: Applying the analytical framework: The working analytical framework was applied by indexing subsequent transcripts, which were single coded by either EW or LH, using the existing categories and codes. EW and LH met regularly to discuss, clarify and expand the developing analytical framework.
- Stage 6: Charting data into the framework matrix: The data were ‘charted’ into a matrix; this involved creating summaries of the data by category from each transcript, identifying illustrative quotations.
- Stage 7: Interpreting the data: EW and LH met regularly to interpret the data through identifying characteristics of and differences between the data. EW and LH also discussed any preliminary impressions, ideas and interpretations of the data which were recorded at an earlier stage of analysis. EW and LH mapped connections between categories (groups of codes) and both between and within cases (participants) to generate a set of themes and sub-themes. Given the paucity of literature on working practically with schools in research, the development of themes and sub-themes was an inductive approach, whereby themes and sub-themes were generated from the accounts of participants rather than pre-existing theory (deductive approach). Themes and sub-themes were then discussed, revised and agreed by all co-authors.
